# Supplementary material for: Natural History and Molecular Characteristics of Korean Patients with Mucopolysaccharidosis Type III
Source: J Pers Med. 2022 Apr 21;12(5):665. doi: 10.3390/jpm12050665 (PMC9145712; doi:10.3390/jpm12050665)
Supplement: Supplementary file 1 [file jpm-12-00665-s001.zip › jpm-1676517-supplementary.pdf]

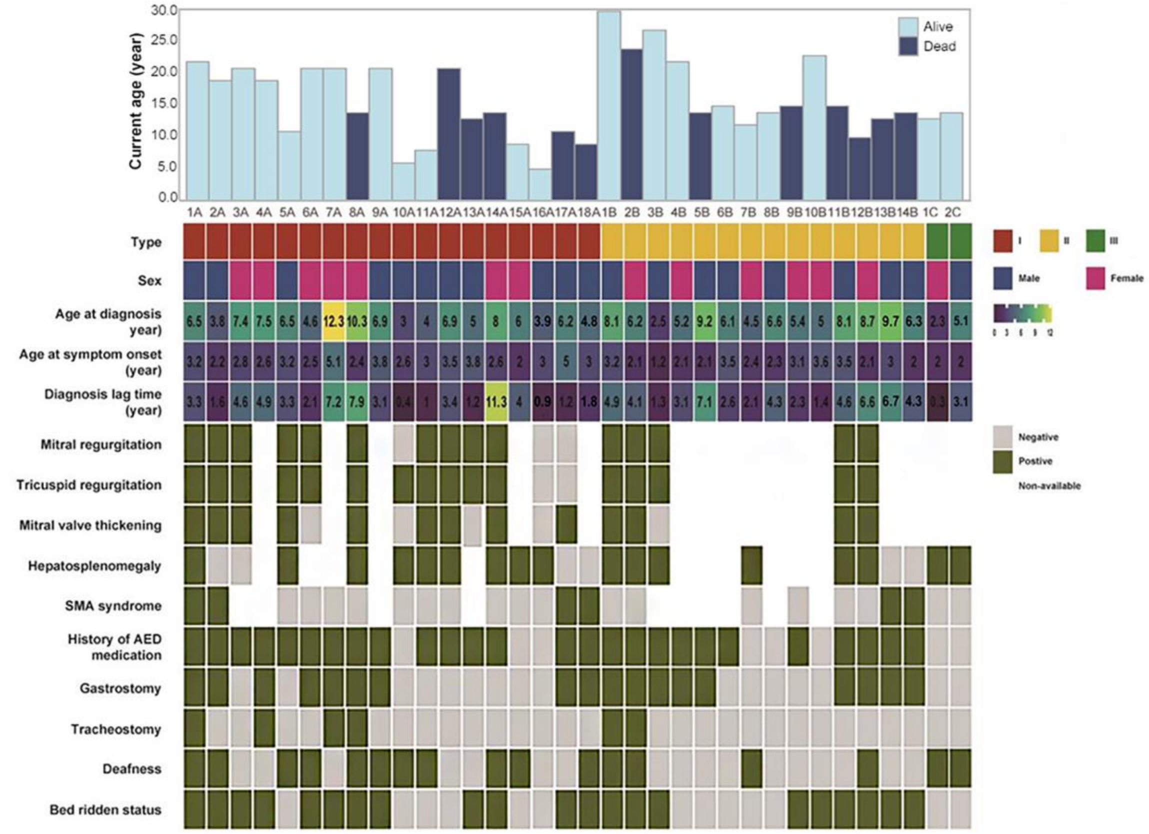

Figure S1. A box plot figure showing the clinical features of all 34 patients.

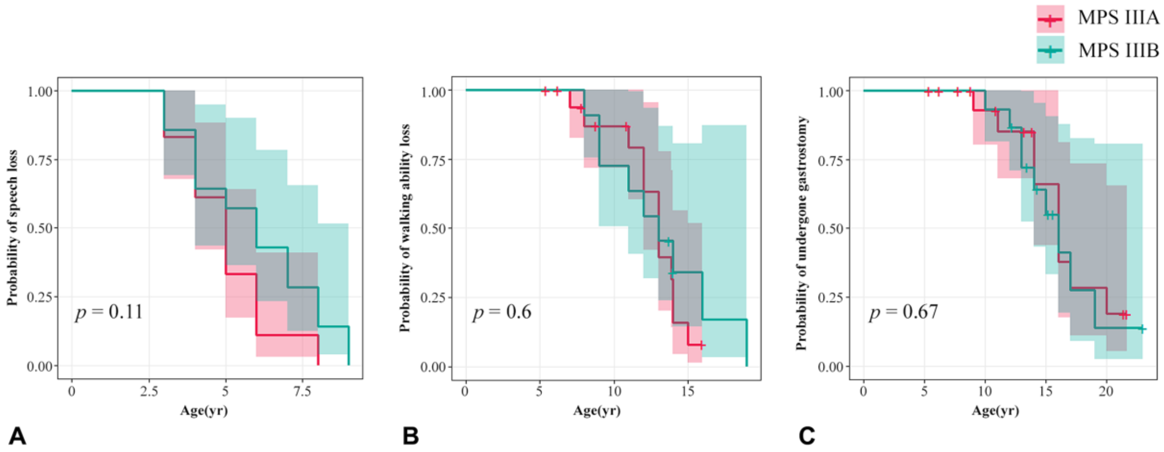

Figure S2. (A–C) Age at speech loss, age at walking ability loss, and age at which gastrostomy was performed in MPS IIIA and IIIB. There were no significant differences between MPS IIIA and IIIB groups when using the log-rank test of the Kaplan–Meier survival curve.
